# Di-μ-perchlorato-bis­{μ-2-[(2-pyrid­yl)methyl­amino­meth­yl]phenolato)dicopper(II) acetonitrile disolvate

**DOI:** 10.1107/S1600536809029134

**Published:** 2009-07-29

**Authors:** Gervas E. Assey, Teshome Yisgedu, Yilma Gultneh, Ray J. Butcher, Yohannes Tesema

**Affiliations:** a Department of Chemistry, Howard University, 525 College Street NW, Washington, DC 20059, USA

## Abstract

In the crystal of the dinuclear title compound, [Cu_2_(C_13_H_13_N_2_O)_2_(ClO_4_)_2_]·2CH_3_CN, the two bridging perchlorate ions chelate to the two Cu^II^ atoms in a μ-*O*:*O*′ fashion on opposite sides of the equatorial plane. The Cu^II^ ions display a distorted octa­hedral coordination geometry (in the usual 4 + 2 Jahn–Teller arrangement), each being coordinated by two O atoms from the two perchlorate ligands, and two N and O atoms from the reduced Schiff base ligand. The asymmetric unit contains two acetonitrile solvent mol­ecules. In the crystal structure, in addition to N—H⋯O hydrogen bonds, there are weak C—H⋯O inter­actions between the perchlorate O atoms and the reduced Schiff base ligand. C—H⋯N inter­actions are also present.

## Related literature

For related structures containing bridging perchlorate anions, see: Sony *et al.* (2006 ▶); Sarkar *et al.* (2004 ▶); Neves *et al.* (2001 ▶); Torelli *et al.* (2000 ▶); O’Connor *et al.* (1986 ▶). For the synthesis, see: Yisgedu (2001 ▶).
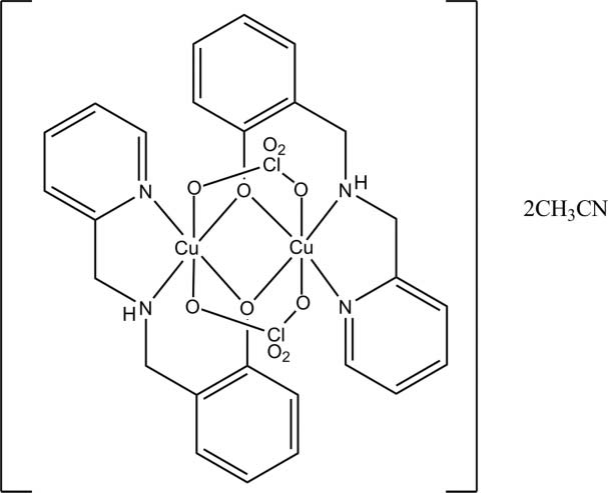


## Experimental

### 

#### Crystal data

[Cu_2_(C_13_H_13_N_2_O)_2_(ClO_4_)_2_]·2C_2_H_3_N
*M* *_r_* = 834.60
Monoclinic, 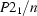

*a* = 16.0285 (4) Å
*b* = 9.4062 (3) Å
*c* = 24.7097 (10) Å
β = 102.665 (3)°
*V* = 3634.8 (2) Å^3^
*Z* = 4
Mo *K*α radiation
μ = 1.38 mm^−1^
*T* = 200 K
0.53 × 0.46 × 0.39 mm

#### Data collection

Oxford Diffraction Gemini diffractometer
Absorption correction: multi-scan (*CrysAlis RED*; Oxford Diffraction, 2008 ▶) *T*_min_ = 0.848, *T*_max_ = 1.000 (expected range = 0.495–0.584)
33853 measured reflections
14212 independent reflections
8089 reflections with *I* > 2σ(*I*)
*R*_int_ = 0.036

#### Refinement

*R*[*F* ^2^ > 2σ(*F* ^2^)] = 0.078
*wR*(*F* ^2^) = 0.199
*S* = 1.19
14212 reflections
453 parameters
6 restraints
H-atom parameters constrained
Δρ_max_ = 1.29 e Å^−3^
Δρ_min_ = −2.24 e Å^−3^

### 

Data collection: *CrysAlis CCD* (Oxford Diffraction, 2008 ▶) ; cell refinement: *CrysAlis RED* (Oxford Diffraction, 2008 ▶); data reduction: *CrysAlis RED*; program(s) used to solve structure: *SHELXS97* (Sheldrick, 2008 ▶); program(s) used to refine structure: *SHELXL97* (Sheldrick, 2008 ▶); molecular graphics: *SHELXTL* (Sheldrick, 2008 ▶); software used to prepare material for publication: *SHELXTL*.

## Supplementary Material

Crystal structure: contains datablocks I, global. DOI: 10.1107/S1600536809029134/ng2609sup1.cif

Structure factors: contains datablocks I. DOI: 10.1107/S1600536809029134/ng2609Isup2.hkl

Additional supplementary materials:  crystallographic information; 3D view; checkCIF report

## Figures and Tables

**Table 1:** Selected bond lengths (Å)

Cu1—O1*B*	1.9410 (18)
Cu1—O1*A*	1.942 (2)
Cu1—N2*A*	1.972 (3)
Cu1—N1*A*	1.974 (2)
Cu1—O21	2.494 (2)
Cu1—O11	2.706 (2)
Cu1—Cu2	2.9543 (5)
Cu2—O1*A*	1.9440 (18)
Cu2—O1*B*	1.952 (2)
Cu2—N2*B*	1.971 (3)
Cu2—N1*B*	1.973 (2)
Cu2—O12	2.489 (2)
Cu2—O22	2.670 (2)

**Table 2:** Hydrogen-bond geometry (Å, °)

*D*—H⋯*A*	*D*—H	H⋯*A*	*D*⋯*A*	*D*—H⋯*A*
N1*A*—H1*AA*⋯O23^i^	0.93	2.05	2.913 (4)	154
N1*B*—H1*BA*⋯O14^ii^	0.93	2.09	2.943 (4)	152
C7*B*—H7*BA*⋯O14^iii^	0.99	2.53	3.167 (4)	122
C22*S*—H22*H*⋯O13^iv^	0.98	2.47	3.264 (8)	138
C7*A*—H7*AB*⋯O23^v^	0.99	2.48	3.143 (4)	124
C4*A*—H4*AA*⋯N1*S*	0.95	2.69	3.643 (7)	175
C12*B*—H12*B*⋯N2*S*	0.95	2.67	3.616 (9)	171

Symmetry codes: (i) 
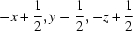
; (ii) 
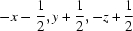
; (iii) 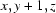; (iv) 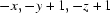; (v) 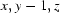.

**Fig. 1.**
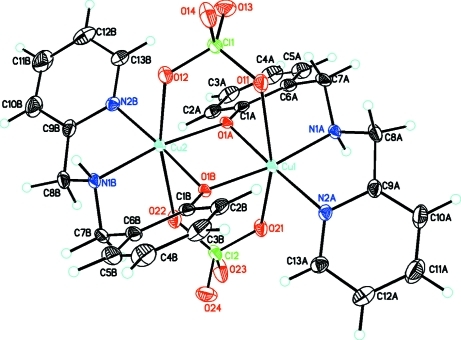
The molecular structure of the dinuclear complex, C26H26Cl2Cu2N4O10 showing the atom numbering scheme and 50% probability displacement ellipsoids.

**Fig. 2.**
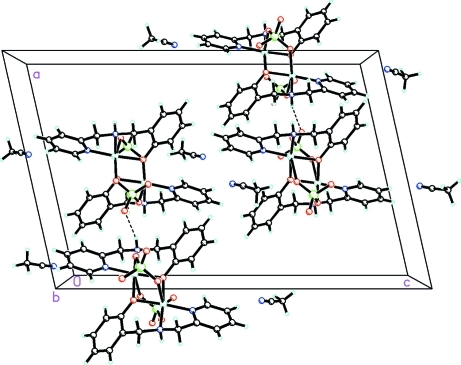
The molecular packing for C26H26Cl2Cu2N4O10, 2(C2H3N) viewed down the b axis showing the intermolecular N—H···O, C—H···O and C–H···N interactions.

## supplementary crystallographic information

### Comment

Dinucleating metal centers in protein complexes play important roles in biology such as dioxygen transport or activation, electron transfer, hydrolytic chemistry and nitrogen oxide reduction (Torelli *et al.* 2000). Many of the copper enzymes have oxidase or oxygenase activities. In order to model dinuclear copper enzymes, copper complexes containing binucleating ligands with bridging phenoxo groups have to be synthesized. In biological systems, type 3 copper enzymes tyrosinase and catechol oxidase contain similar dinuclear copper active sites and are responsible for hydroxylation of monophenols and/or oxidation of catechols (Neves *et al.* 2001).

We report here the synthesis and crystal structure determination of binuclear copper complex C_30_H_32_Cl_2_Cu_2_N_6_O_10_ for which the molecular structure is shown in Fig.1. The geometry around the Cu^II^ ions in Fig. 1 of the title compound can be described as that of distorted octahedral (with the usual tetragonal distortion seen in 6-coordinate Cu complexes) with each Cu^II^ ion coordinated to one pyridine N atom, a secondary amine N atom, two O atoms from the perchlorate groups and two bridging O atoms from the salicylaldamine groups. The Cu—N_py_ bond distances are; Cu(1)—N(2 A) 1.973 (3) Å and Cu(2)—N(2B) 1.971 (3) Å. The Cu—N_amine_ bond distances are: Cu(1)—N(1 A) 1.975 (2) Å and Cu(2)—N(1B) 1.974 (2) Å. Having two copper centers bridged by a perchlorate ion has been previously observed (Sony *et al.*, 2006; O'Connor *et al.*, 1986). However, in this instance, the two copper centers are bridged by two perchlorate ions on opposite sides of the equatorial plane. The Cu—O_perchlorate_ distances range from 2.489 (2) to 2.707 (3) Å. The Cu—Cu distance is 2.9542 (5) Å.

The crystals contain two molecules of acetonitrile as solvate. In the crystal structure, in addition to N—H···O hydrogen bonds, there are C—H···O interactions between the perchlorate O atoms and the reduced Schiff base ligand.

### Experimental

The ligand (2-pyridylmethyl)(2-hydroxybenzyl)amine (*L*^1^H) was synthesized as described below (Yisgedu, 2001). To 5.4 g (50 mmol) of 2-(2-aminomethyl)pyridine in 10 ml of ethanol was added 6.1 g (50 mmol) of salicylaldehyde in 15 ml of ethanol which resulted in a deep yellow color. The solution was left to stir for 30 minutes. A sodium borohydride solution (3 g NaBH_4_, 0.4 g NaOH, and 40.0 ml of H_2_O) were added dropwise. The solution changed to colorless and was left to stir for one hour after adding all the NaBH_4_ solution. The volume of the solution was reduced to 20 ml after extracting three times with chloroform (3 *x* 40 ml). The extracts were combined and dried in anhydrous Na_2_SO_4_ overnight. The Na_2_SO_4_ was filtered and the filtrate concentrated to give a colorless oil (9.3 g, 87%).

The metal complex was synthesized as described below (Yisgedu, 2001). 1.64 g (7.65 mmol) of *L*^1^H was mixed with 2.86 g (7.65 mmol) of Cu(ClO_4_)_2_.6H_2_O in 25 ml MeOH and 1.75 ml NaOCH_3_. The solution mixture was stirred overnight, filtered, washed with 1:1 mixture of ether and methanol and dried to give 2.3 g (80%). Crystals suitable for X-ray diffraction were obtained by layering a solution of the complex in acetonitrile with diethyl ether.

### Refinement

H atoms were placed in geometrically idealized positions and constrained to ride on their parent atoms with a C—H distance of 0.95 and 0.99 Å *U*_iso_(H) = 1.2*U*_eq_(C). The H atoms attached to N were idealized with an N–H distance of 0.93 Å. The highest peak and lowest peaks in the final difference Fourier were 1.29 and -2.24 e/Å^3^ [0.71 and 0.70 Å from Cu1].

### Crystal data

**Table:**

[Cu_2_(C_13_H_13_N_2_O)_2_(ClO_4_)_2_]·2C_2_H_3_N	*F*(000) = 1704
*M**_r_* = 834.60	*D*_x_ = 1.525 Mg m^−^^3^
Monoclinic, *P*2_1_/*n*	Mo *K*α radiation, λ = 0.71073 Å
*a* = 16.0285 (4) Å	Cell parameters from 11642 reflections
*b* = 9.4062 (3) Å	θ = 4.7–34.7°
*c* = 24.7097 (10) Å	µ = 1.38 mm^−^^1^
β = 102.665 (3)°	*T* = 200 K
*V* = 3634.8 (2) Å^3^	Chunk, dark green
*Z* = 4	0.53 × 0.46 × 0.39 mm

### Data collection

**Table:**

Oxford Diffraction Gemini diffractometer	14212 independent reflections
Radiation source: fine-focus sealed tube	8089 reflections with *I* > 2σ(*I*)
graphite	*R*_int_ = 0.036
Detector resolution: 10.5081 pixels mm^-1^	θ_max_ = 34.8°, θ_min_ = 4.7°
ω scans	*h* = −25→25
Absorption correction: multi-scan (*CrysAlis RED*; Oxford Diffraction, 2008)	*k* = −14→11
*T*_min_ = 0.848, *T*_max_ = 1.000	*l* = −39→38
33853 measured reflections	

### Refinement

**Table:**

Refinement on *F*^2^	Primary atom site location: structure-invariant direct methods
Least-squares matrix: full	Secondary atom site location: difference Fourier map
*R*[*F*^2^ > 2σ(*F*^2^)] = 0.078	Hydrogen site location: inferred from neighbouring sites
*wR*(*F*^2^) = 0.199	H-atom parameters constrained
*S* = 1.19	*w* = 1/[σ^2^(*F*_o_^2^) + (0.0713*P*)^2^ + 3.514*P*] where *P* = (*F*_o_^2^ + 2*F*_c_^2^)/3
14212 reflections	(Δ/σ)_max_ = 0.002
453 parameters	Δρ_max_ = 1.29 e Å^−^^3^
6 restraints	Δρ_min_ = −2.24 e Å^−^^3^

### Special details

**Table:**

Experimental. CrysAlis RED, Oxford Diffraction Ltd., Version 1.171.32.15 (release 10-01-2008 CrysAlis171 .NET) (compiled Jan 10 2008,16:37:18) Empirical absorption correction using spherical harmonics, implemented in SCALE3 ABSPACK scaling algorithm (Oxford Diffraction, 2008).
Geometry. All e.s.d.'s (except the e.s.d. in the dihedral angle between two l.s. planes) are estimated using the full covariance matrix. The cell e.s.d.'s are taken into account individually in the estimation of e.s.d.'s in distances, angles and torsion angles; correlations between e.s.d.'s in cell parameters are only used when they are defined by crystal symmetry. An approximate (isotropic) treatment of cell e.s.d.'s is used for estimating e.s.d.'s involving l.s. planes.
Refinement. Refinement of *F*^2^ against ALL reflections. The weighted *R*-factor *wR* and goodness of fit *S* are based on *F*^2^, conventional *R*-factors *R* are based on *F*, with *F* set to zero for negative *F*^2^. The threshold expression of *F*^2^ > σ(*F*^2^) is used only for calculating *R*-factors(gt) *etc*. and is not relevant to the choice of reflections for refinement. *R*-factors based on *F*^2^ are statistically about twice as large as those based on *F*, and *R*- factors based on ALL data will be even larger.

### Fractional atomic coordinates and isotropic or equivalent isotropic displacement parameters (Å^2^)

**Table:**

	*x*	*y*	*z*	*U*_iso_*/*U*_eq_	
Cu1	0.05889 (2)	0.91878 (4)	0.214778 (15)	0.02910 (8)	
Cu2	−0.06076 (2)	1.04142 (4)	0.277641 (15)	0.02901 (8)	
Cl1	−0.10838 (4)	0.68198 (8)	0.24709 (4)	0.03933 (18)	
Cl2	0.10650 (4)	1.27847 (8)	0.24782 (4)	0.0440 (2)	
O11	−0.05145 (14)	0.6989 (3)	0.20982 (10)	0.0450 (6)	
O12	−0.14408 (13)	0.8171 (2)	0.25739 (10)	0.0425 (6)	
O13	−0.06250 (19)	0.6237 (3)	0.29860 (13)	0.0743 (9)	
O14	−0.17758 (15)	0.5894 (3)	0.22222 (15)	0.0730 (10)	
O21	0.14267 (14)	1.1426 (2)	0.23742 (11)	0.0443 (6)	
O22	0.04757 (14)	1.2593 (3)	0.28331 (11)	0.0483 (6)	
O23	0.17503 (16)	1.3695 (3)	0.27486 (17)	0.0819 (11)	
O24	0.0624 (2)	1.3376 (4)	0.19615 (15)	0.0843 (10)	
O1A	0.04239 (11)	0.9262 (2)	0.29027 (8)	0.0264 (4)	
O1B	−0.04444 (11)	1.0330 (2)	0.20172 (8)	0.0262 (4)	
N1A	0.15385 (15)	0.7811 (3)	0.23295 (11)	0.0347 (6)	
H1AA	0.2049	0.8316	0.2381	0.042*	
N2A	0.08589 (15)	0.9092 (3)	0.14074 (11)	0.0332 (6)	
N1B	−0.15526 (15)	1.1796 (3)	0.25897 (11)	0.0347 (6)	
H1BA	−0.2064	1.1300	0.2551	0.042*	
N2B	−0.08647 (15)	1.0555 (3)	0.35188 (10)	0.0333 (6)	
C1A	0.11031 (17)	0.9121 (3)	0.33359 (12)	0.0328 (6)	
C2A	0.1249 (2)	1.0062 (4)	0.37820 (13)	0.0432 (8)	
H2AA	0.0877	1.0847	0.3784	0.052*	
C3A	0.1937 (2)	0.9851 (5)	0.42218 (15)	0.0599 (11)	
H3AA	0.2019	1.0478	0.4530	0.072*	
C4A	0.2502 (3)	0.8764 (6)	0.42243 (17)	0.0730 (14)	
H4AA	0.2973	0.8640	0.4529	0.088*	
C5A	0.2379 (2)	0.7856 (5)	0.37814 (18)	0.0617 (12)	
H5AA	0.2780	0.7114	0.3779	0.074*	
C6A	0.16741 (19)	0.7989 (4)	0.33285 (14)	0.0408 (8)	
C7A	0.15409 (19)	0.7000 (3)	0.28532 (15)	0.0441 (8)	
H7AA	0.0989	0.6498	0.2820	0.053*	
H7AB	0.2003	0.6281	0.2914	0.053*	
C8A	0.1471 (2)	0.6924 (3)	0.18345 (16)	0.0449 (8)	
H8AA	0.0998	0.6232	0.1807	0.054*	
H8AB	0.2009	0.6392	0.1852	0.054*	
C9A	0.1302 (2)	0.7899 (4)	0.13437 (15)	0.0422 (8)	
C10A	0.1570 (3)	0.7668 (5)	0.08580 (19)	0.0721 (13)	
H10A	0.1873	0.6826	0.0810	0.087*	
C11A	0.1395 (3)	0.8674 (6)	0.0441 (2)	0.0800 (14)	
H11A	0.1568	0.8517	0.0102	0.096*	
C12A	0.0973 (3)	0.9893 (5)	0.05174 (16)	0.0597 (11)	
H12A	0.0867	1.0606	0.0238	0.072*	
C13A	0.0704 (2)	1.0075 (4)	0.10056 (14)	0.0414 (8)	
H13A	0.0402	1.0915	0.1058	0.050*	
C1B	−0.11278 (17)	1.0433 (3)	0.15872 (12)	0.0329 (6)	
C2B	−0.1271 (2)	0.9480 (4)	0.11472 (13)	0.0404 (8)	
H2BA	−0.0887	0.8711	0.1146	0.048*	
C3B	−0.1972 (2)	0.9648 (5)	0.07100 (15)	0.0583 (11)	
H3BA	−0.2054	0.9006	0.0406	0.070*	
C4B	−0.2548 (3)	1.0718 (6)	0.07082 (17)	0.0659 (13)	
H4BA	−0.3028	1.0817	0.0408	0.079*	
C5B	−0.2424 (2)	1.1645 (5)	0.11447 (17)	0.0593 (11)	
H5BA	−0.2833	1.2375	0.1147	0.071*	
C6B	−0.17098 (19)	1.1552 (4)	0.15908 (14)	0.0417 (8)	
C7B	−0.1574 (2)	1.2582 (3)	0.20605 (15)	0.0413 (8)	
H7BA	−0.2043	1.3288	0.1998	0.050*	
H7BB	−0.1028	1.3095	0.2084	0.050*	
C8B	−0.1461 (2)	1.2726 (3)	0.30813 (15)	0.0430 (8)	
H8BA	−0.1995	1.3268	0.3068	0.052*	
H8BB	−0.0987	1.3409	0.3094	0.052*	
C9B	−0.1276 (2)	1.1781 (4)	0.35779 (15)	0.0433 (8)	
C10B	−0.1509 (3)	1.2083 (5)	0.40691 (19)	0.0734 (13)	
H10B	−0.1796	1.2946	0.4112	0.088*	
C11B	−0.1321 (4)	1.1125 (6)	0.4497 (2)	0.0852 (16)	
H11B	−0.1458	1.1342	0.4842	0.102*	
C12B	−0.0936 (3)	0.9851 (6)	0.44275 (17)	0.0684 (13)	
H12B	−0.0829	0.9162	0.4715	0.082*	
C13B	−0.0707 (2)	0.9600 (4)	0.39270 (14)	0.0473 (9)	
H13B	−0.0433	0.8732	0.3874	0.057*	
N1S	0.4394 (4)	0.8308 (7)	0.5336 (2)	0.129 (2)	
C11S	0.4318 (6)	0.9186 (8)	0.5605 (3)	0.124 (3)	
C12S	0.4157 (9)	1.0326 (9)	0.5941 (3)	0.191 (5)	
H12C	0.4518	1.1138	0.5895	0.287*	
H12D	0.4287	1.0028	0.6331	0.287*	
H12E	0.3554	1.0602	0.5831	0.287*	
N2S	−0.0703 (6)	0.6977 (8)	0.5421 (3)	0.175 (3)	
C21S	−0.0794 (5)	0.6218 (9)	0.5687 (2)	0.125 (3)	
C22S	−0.0856 (7)	0.5063 (9)	0.6015 (3)	0.159 (4)	
H22F	−0.0700	0.4201	0.5837	0.238*	
H22G	−0.1444	0.4976	0.6063	0.238*	
H22H	−0.0468	0.5185	0.6378	0.238*	

### Atomic displacement parameters (Å^2^)

**Table:**

	*U*^11^	*U*^22^	*U*^33^	*U*^12^	*U*^13^	*U*^23^
Cu1	0.02109 (14)	0.02713 (17)	0.03988 (18)	0.00594 (13)	0.00843 (13)	0.00543 (14)
Cu2	0.02246 (15)	0.02678 (17)	0.03891 (18)	0.00478 (13)	0.00915 (13)	0.00460 (14)
Cl1	0.0237 (3)	0.0266 (3)	0.0693 (5)	−0.0011 (3)	0.0136 (3)	0.0037 (3)
Cl2	0.0232 (3)	0.0261 (3)	0.0851 (6)	−0.0005 (3)	0.0166 (3)	0.0049 (4)
O11	0.0344 (11)	0.0397 (12)	0.0651 (15)	0.0012 (10)	0.0203 (11)	−0.0048 (11)
O12	0.0290 (10)	0.0281 (11)	0.0729 (15)	−0.0041 (9)	0.0165 (10)	−0.0073 (10)
O13	0.0604 (17)	0.079 (2)	0.0856 (19)	0.0134 (15)	0.0201 (15)	0.0459 (16)
O14	0.0306 (11)	0.0388 (14)	0.153 (3)	−0.0105 (10)	0.0276 (15)	−0.0308 (16)
O21	0.0327 (10)	0.0316 (11)	0.0726 (15)	−0.0027 (9)	0.0198 (11)	−0.0094 (11)
O22	0.0330 (11)	0.0421 (13)	0.0728 (16)	−0.0001 (10)	0.0181 (11)	−0.0070 (12)
O23	0.0329 (12)	0.0408 (14)	0.174 (3)	−0.0134 (11)	0.0275 (16)	−0.0393 (17)
O24	0.0676 (19)	0.081 (2)	0.110 (2)	0.0226 (16)	0.0311 (17)	0.0583 (18)
O1A	0.0194 (8)	0.0242 (9)	0.0333 (9)	0.0033 (7)	0.0009 (7)	0.0083 (7)
O1B	0.0179 (8)	0.0263 (9)	0.0325 (9)	0.0049 (7)	0.0016 (7)	0.0068 (7)
N1A	0.0231 (10)	0.0268 (12)	0.0568 (15)	0.0031 (9)	0.0141 (11)	0.0034 (11)
N2A	0.0239 (10)	0.0333 (13)	0.0444 (13)	−0.0014 (10)	0.0117 (10)	−0.0027 (11)
N1B	0.0215 (10)	0.0281 (12)	0.0563 (15)	0.0017 (9)	0.0124 (10)	0.0035 (11)
N2B	0.0287 (11)	0.0344 (13)	0.0374 (12)	−0.0059 (10)	0.0084 (10)	−0.0017 (10)
C1A	0.0208 (11)	0.0384 (15)	0.0387 (14)	−0.0040 (11)	0.0054 (11)	0.0161 (12)
C2A	0.0340 (15)	0.055 (2)	0.0394 (16)	−0.0127 (15)	0.0065 (13)	0.0070 (14)
C3A	0.0455 (19)	0.091 (3)	0.0385 (18)	−0.022 (2)	−0.0004 (16)	0.0096 (19)
C4A	0.042 (2)	0.116 (4)	0.050 (2)	−0.011 (2)	−0.0129 (18)	0.028 (2)
C5A	0.0308 (16)	0.078 (3)	0.071 (2)	0.0094 (18)	−0.0007 (17)	0.036 (2)
C6A	0.0263 (13)	0.0437 (17)	0.0500 (17)	0.0037 (13)	0.0034 (13)	0.0219 (14)
C7A	0.0256 (13)	0.0325 (15)	0.074 (2)	0.0097 (12)	0.0116 (14)	0.0224 (15)
C8A	0.0322 (15)	0.0291 (15)	0.076 (2)	0.0055 (13)	0.0183 (16)	−0.0042 (15)
C9A	0.0342 (14)	0.0363 (17)	0.061 (2)	−0.0026 (13)	0.0206 (14)	−0.0079 (15)
C10A	0.083 (3)	0.063 (3)	0.086 (3)	0.007 (2)	0.052 (2)	−0.017 (2)
C11A	0.100 (3)	0.085 (3)	0.069 (3)	−0.006 (3)	0.051 (2)	−0.016 (2)
C12A	0.062 (2)	0.072 (3)	0.049 (2)	−0.016 (2)	0.0207 (18)	0.0019 (19)
C13A	0.0349 (15)	0.0461 (18)	0.0434 (17)	−0.0056 (14)	0.0093 (13)	0.0036 (14)
C1B	0.0227 (12)	0.0354 (15)	0.0401 (14)	−0.0012 (11)	0.0056 (11)	0.0160 (12)
C2B	0.0304 (14)	0.0536 (19)	0.0375 (15)	−0.0065 (14)	0.0081 (12)	0.0085 (14)
C3B	0.0408 (18)	0.094 (3)	0.0380 (17)	−0.014 (2)	0.0050 (15)	0.0084 (19)
C4B	0.0387 (19)	0.104 (3)	0.048 (2)	0.001 (2)	−0.0067 (16)	0.023 (2)
C5B	0.0313 (16)	0.076 (3)	0.067 (2)	0.0151 (17)	0.0027 (16)	0.035 (2)
C6B	0.0260 (13)	0.0445 (18)	0.0539 (18)	0.0072 (13)	0.0074 (13)	0.0220 (15)
C7B	0.0300 (14)	0.0312 (15)	0.064 (2)	0.0091 (12)	0.0140 (14)	0.0167 (14)
C8B	0.0357 (15)	0.0305 (16)	0.067 (2)	0.0030 (13)	0.0192 (15)	−0.0096 (14)
C9B	0.0326 (14)	0.0417 (18)	0.060 (2)	−0.0050 (13)	0.0195 (14)	−0.0122 (15)
C10B	0.085 (3)	0.068 (3)	0.081 (3)	0.000 (2)	0.047 (2)	−0.021 (2)
C11B	0.113 (4)	0.089 (4)	0.068 (3)	−0.009 (3)	0.052 (3)	−0.021 (3)
C12B	0.076 (3)	0.087 (3)	0.046 (2)	−0.026 (3)	0.022 (2)	0.003 (2)
C13B	0.0449 (18)	0.053 (2)	0.0460 (18)	−0.0111 (16)	0.0133 (15)	0.0022 (16)
N1S	0.159 (6)	0.107 (4)	0.106 (4)	0.031 (4)	−0.002 (4)	−0.003 (3)
C11S	0.171 (7)	0.097 (5)	0.077 (4)	0.021 (5)	−0.034 (4)	−0.004 (3)
C12S	0.354 (15)	0.108 (6)	0.093 (5)	0.047 (8)	0.011 (7)	−0.014 (5)
N2S	0.225 (8)	0.136 (6)	0.135 (6)	−0.078 (6)	−0.022 (5)	0.019 (5)
C21S	0.160 (6)	0.125 (5)	0.063 (3)	−0.062 (5)	−0.035 (4)	0.025 (3)
C22S	0.221 (10)	0.130 (6)	0.099 (5)	−0.043 (6)	−0.022 (6)	0.044 (5)

### Geometric parameters (Å, °)

**Table:**

Cu1—O1B	1.9410 (18)	C8A—C9A	1.497 (5)
Cu1—O1A	1.942 (2)	C8A—H8AA	0.9900
Cu1—N2A	1.972 (3)	C8A—H8AB	0.9900
Cu1—N1A	1.974 (2)	C9A—C10A	1.378 (6)
Cu1—O21	2.494 (2)	C10A—C11A	1.381 (7)
Cu1—O11	2.706 (2)	C10A—H10A	0.9500
Cu1—Cu2	2.9543 (5)	C11A—C12A	1.365 (7)
Cu2—O1A	1.9440 (18)	C11A—H11A	0.9500
Cu2—O1B	1.952 (2)	C12A—C13A	1.378 (5)
Cu2—N2B	1.971 (3)	C12A—H12A	0.9500
Cu2—N1B	1.973 (2)	C13A—H13A	0.9500
Cu2—O12	2.489 (2)	C1B—C2B	1.389 (5)
Cu2—O22	2.670 (2)	C1B—C6B	1.408 (4)
Cl1—O13	1.432 (3)	C2B—C3B	1.387 (4)
Cl1—O14	1.438 (3)	C2B—H2BA	0.9500
Cl1—O12	1.439 (2)	C3B—C4B	1.365 (6)
Cl1—O11	1.440 (3)	C3B—H3BA	0.9500
Cl2—O24	1.429 (3)	C4B—C5B	1.367 (6)
Cl2—O22	1.434 (3)	C4B—H4BA	0.9500
Cl2—O23	1.437 (3)	C5B—C6B	1.407 (5)
Cl2—O21	1.449 (2)	C5B—H5BA	0.9500
O1A—C1A	1.356 (3)	C6B—C7B	1.490 (5)
O1B—C1B	1.352 (3)	C7B—H7BA	0.9900
N1A—C8A	1.465 (4)	C7B—H7BB	0.9900
N1A—C7A	1.502 (4)	C8B—C9B	1.492 (5)
N1A—H1AA	0.9300	C8B—H8BA	0.9900
N2A—C13A	1.339 (4)	C8B—H8BB	0.9900
N2A—C9A	1.355 (4)	C9B—C10B	1.375 (6)
N1B—C8B	1.478 (4)	C10B—C11B	1.371 (7)
N1B—C7B	1.496 (4)	C10B—H10B	0.9500
N1B—H1BA	0.9300	C11B—C12B	1.376 (7)
N2B—C13B	1.333 (4)	C11B—H11B	0.9500
N2B—C9B	1.352 (4)	C12B—C13B	1.385 (6)
C1A—C2A	1.393 (5)	C12B—H12B	0.9500
C1A—C6A	1.407 (4)	C13B—H13B	0.9500
C2A—C3A	1.383 (5)	N1S—C11S	1.083 (8)
C2A—H2AA	0.9500	C11S—C12S	1.416 (11)
C3A—C4A	1.364 (7)	C12S—H12C	0.9800
C3A—H3AA	0.9500	C12S—H12D	0.9800
C4A—C5A	1.368 (7)	C12S—H12E	0.9800
C4A—H4AA	0.9500	N2S—C21S	1.003 (9)
C5A—C6A	1.410 (5)	C21S—C22S	1.371 (10)
C5A—H5AA	0.9500	C22S—H22F	0.9800
C6A—C7A	1.476 (5)	C22S—H22G	0.9800
C7A—H7AA	0.9900	C22S—H22H	0.9800
C7A—H7AB	0.9900		
			
O1B—Cu1—O1A	81.30 (8)	C5A—C4A—H4AA	120.5
O1B—Cu1—N2A	102.94 (9)	C4A—C5A—C6A	122.0 (4)
O1A—Cu1—N2A	175.24 (8)	C4A—C5A—H5AA	119.0
O1B—Cu1—N1A	171.26 (9)	C6A—C5A—H5AA	119.0
O1A—Cu1—N1A	93.77 (10)	C1A—C6A—C5A	117.8 (3)
N2A—Cu1—N1A	82.30 (11)	C1A—C6A—C7A	120.4 (3)
O1B—Cu1—O21	88.16 (7)	C5A—C6A—C7A	121.8 (3)
O1A—Cu1—O21	86.22 (8)	C6A—C7A—N1A	109.7 (3)
N2A—Cu1—O21	91.71 (9)	C6A—C7A—H7AA	109.7
N1A—Cu1—O21	98.77 (9)	N1A—C7A—H7AA	109.7
O1B—Cu1—O11	83.69 (7)	C6A—C7A—H7AB	109.7
O1A—Cu1—O11	81.12 (8)	N1A—C7A—H7AB	109.7
N2A—Cu1—O11	101.38 (9)	H7AA—C7A—H7AB	108.2
N1A—Cu1—O11	88.43 (9)	N1A—C8A—C9A	107.1 (3)
O21—Cu1—O11	165.84 (8)	N1A—C8A—H8AA	110.3
O1B—Cu1—Cu2	40.76 (6)	C9A—C8A—H8AA	110.3
O1A—Cu1—Cu2	40.54 (5)	N1A—C8A—H8AB	110.3
N2A—Cu1—Cu2	143.65 (7)	C9A—C8A—H8AB	110.3
N1A—Cu1—Cu2	133.92 (8)	H8AA—C8A—H8AB	108.5
O21—Cu1—Cu2	86.10 (6)	N2A—C9A—C10A	120.2 (4)
O11—Cu1—Cu2	80.16 (5)	N2A—C9A—C8A	114.8 (3)
O1A—Cu2—O1B	80.98 (8)	C10A—C9A—C8A	125.0 (3)
O1A—Cu2—N2B	103.41 (9)	C9A—C10A—C11A	119.4 (4)
O1B—Cu2—N2B	175.47 (9)	C9A—C10A—H10A	120.3
O1A—Cu2—N1B	170.89 (10)	C11A—C10A—H10A	120.3
O1B—Cu2—N1B	93.43 (10)	C12A—C11A—C10A	119.9 (4)
N2B—Cu2—N1B	82.36 (11)	C12A—C11A—H11A	120.0
O1A—Cu2—O12	87.68 (7)	C10A—C11A—H11A	120.0
O1B—Cu2—O12	87.22 (8)	C11A—C12A—C13A	119.0 (4)
N2B—Cu2—O12	91.81 (9)	C11A—C12A—H12A	120.5
N1B—Cu2—O12	99.28 (9)	C13A—C12A—H12A	120.5
O1A—Cu2—O22	84.20 (7)	N2A—C13A—C12A	121.4 (4)
O1B—Cu2—O22	81.88 (8)	N2A—C13A—H13A	119.3
N2B—Cu2—O22	99.55 (9)	C12A—C13A—H13A	119.3
N1B—Cu2—O22	87.91 (9)	O1B—C1B—C2B	122.4 (3)
O12—Cu2—O22	167.30 (8)	O1B—C1B—C6B	118.2 (3)
O1A—Cu2—Cu1	40.49 (6)	C2B—C1B—C6B	119.5 (3)
O1B—Cu2—Cu1	40.49 (5)	C3B—C2B—C1B	120.2 (4)
N2B—Cu2—Cu1	143.89 (7)	C3B—C2B—H2BA	119.9
N1B—Cu2—Cu1	133.48 (8)	C1B—C2B—H2BA	119.9
O12—Cu2—Cu1	86.83 (6)	C4B—C3B—C2B	121.2 (4)
O22—Cu2—Cu1	80.65 (5)	C4B—C3B—H3BA	119.4
O13—Cl1—O14	110.2 (2)	C2B—C3B—H3BA	119.4
O13—Cl1—O12	109.12 (18)	C3B—C4B—C5B	119.1 (3)
O14—Cl1—O12	108.11 (14)	C3B—C4B—H4BA	120.4
O13—Cl1—O11	109.68 (16)	C5B—C4B—H4BA	120.4
O14—Cl1—O11	109.34 (18)	C4B—C5B—C6B	122.1 (4)
O12—Cl1—O11	110.34 (14)	C4B—C5B—H5BA	119.0
O24—Cl2—O22	109.42 (17)	C6B—C5B—H5BA	119.0
O24—Cl2—O23	111.2 (2)	C5B—C6B—C1B	117.9 (3)
O22—Cl2—O23	109.24 (19)	C5B—C6B—C7B	121.6 (3)
O24—Cl2—O21	108.87 (19)	C1B—C6B—C7B	120.5 (3)
O22—Cl2—O21	109.87 (15)	C6B—C7B—N1B	109.4 (3)
O23—Cl2—O21	108.26 (14)	C6B—C7B—H7BA	109.8
Cl1—O11—Cu1	123.70 (13)	N1B—C7B—H7BA	109.8
Cl1—O12—Cu2	124.58 (12)	C6B—C7B—H7BB	109.8
Cl2—O21—Cu1	124.58 (12)	N1B—C7B—H7BB	109.8
Cl2—O22—Cu2	124.93 (14)	H7BA—C7B—H7BB	108.3
C1A—O1A—Cu1	120.01 (18)	N1B—C8B—C9B	106.8 (3)
C1A—O1A—Cu2	133.53 (19)	N1B—C8B—H8BA	110.4
Cu1—O1A—Cu2	98.98 (8)	C9B—C8B—H8BA	110.4
C1B—O1B—Cu1	133.25 (19)	N1B—C8B—H8BB	110.4
C1B—O1B—Cu2	119.75 (17)	C9B—C8B—H8BB	110.4
Cu1—O1B—Cu2	98.74 (8)	H8BA—C8B—H8BB	108.6
C8A—N1A—C7A	114.6 (3)	N2B—C9B—C10B	120.3 (4)
C8A—N1A—Cu1	105.76 (18)	N2B—C9B—C8B	115.7 (3)
C7A—N1A—Cu1	112.68 (19)	C10B—C9B—C8B	124.0 (3)
C8A—N1A—H1AA	107.9	C11B—C10B—C9B	119.2 (4)
C7A—N1A—H1AA	107.9	C11B—C10B—H10B	120.4
Cu1—N1A—H1AA	107.9	C9B—C10B—H10B	120.4
C13A—N2A—C9A	120.2 (3)	C10B—C11B—C12B	120.4 (4)
C13A—N2A—Cu1	127.8 (2)	C10B—C11B—H11B	119.8
C9A—N2A—Cu1	111.9 (2)	C12B—C11B—H11B	119.8
C8B—N1B—C7B	113.9 (3)	C11B—C12B—C13B	118.2 (4)
C8B—N1B—Cu2	105.36 (18)	C11B—C12B—H12B	120.9
C7B—N1B—Cu2	113.53 (19)	C13B—C12B—H12B	120.9
C8B—N1B—H1BA	107.9	N2B—C13B—C12B	121.2 (4)
C7B—N1B—H1BA	107.9	N2B—C13B—H13B	119.4
Cu2—N1B—H1BA	107.9	C12B—C13B—H13B	119.4
C13B—N2B—C9B	120.6 (3)	N1S—C11S—C12S	176.0 (11)
C13B—N2B—Cu2	128.0 (2)	C11S—C12S—H12C	109.5
C9B—N2B—Cu2	111.4 (2)	C11S—C12S—H12D	109.5
O1A—C1A—C2A	122.0 (3)	H12C—C12S—H12D	109.5
O1A—C1A—C6A	118.4 (3)	C11S—C12S—H12E	109.5
C2A—C1A—C6A	119.7 (3)	H12C—C12S—H12E	109.5
C3A—C2A—C1A	119.8 (4)	H12D—C12S—H12E	109.5
C3A—C2A—H2AA	120.1	N2S—C21S—C22S	172.2 (12)
C1A—C2A—H2AA	120.1	C21S—C22S—H22F	109.5
C4A—C3A—C2A	121.7 (4)	C21S—C22S—H22G	109.5
C4A—C3A—H3AA	119.1	H22F—C22S—H22G	109.5
C2A—C3A—H3AA	119.1	C21S—C22S—H22H	109.5
C3A—C4A—C5A	119.0 (3)	H22F—C22S—H22H	109.5
C3A—C4A—H4AA	120.5	H22G—C22S—H22H	109.5
			
O1B—Cu1—Cu2—O1A	179.56 (12)	O1A—Cu1—N1A—C8A	146.8 (2)
N2A—Cu1—Cu2—O1A	−176.33 (15)	N2A—Cu1—N1A—C8A	−35.8 (2)
N1A—Cu1—Cu2—O1A	9.69 (13)	O21—Cu1—N1A—C8A	−126.4 (2)
O21—Cu1—Cu2—O1A	−88.74 (10)	O11—Cu1—N1A—C8A	65.9 (2)
O11—Cu1—Cu2—O1A	87.82 (10)	Cu2—Cu1—N1A—C8A	140.56 (17)
O1A—Cu1—Cu2—O1B	−179.56 (12)	O1B—Cu1—N1A—C7A	−34.4 (7)
N2A—Cu1—Cu2—O1B	4.11 (15)	O1A—Cu1—N1A—C7A	21.0 (2)
N1A—Cu1—Cu2—O1B	−169.88 (13)	N2A—Cu1—N1A—C7A	−161.7 (2)
O21—Cu1—Cu2—O1B	91.70 (10)	O21—Cu1—N1A—C7A	107.7 (2)
O11—Cu1—Cu2—O1B	−91.74 (10)	O11—Cu1—N1A—C7A	−60.0 (2)
O1B—Cu1—Cu2—N2B	178.15 (15)	Cu2—Cu1—N1A—C7A	14.7 (2)
O1A—Cu1—Cu2—N2B	−1.41 (15)	O1B—Cu1—N2A—C13A	31.6 (3)
N2A—Cu1—Cu2—N2B	−177.74 (17)	O1A—Cu1—N2A—C13A	−121.0 (11)
N1A—Cu1—Cu2—N2B	8.28 (16)	N1A—Cu1—N2A—C13A	−155.5 (3)
O21—Cu1—Cu2—N2B	−90.15 (14)	O21—Cu1—N2A—C13A	−56.9 (3)
O11—Cu1—Cu2—N2B	86.41 (13)	O11—Cu1—N2A—C13A	117.7 (2)
O1B—Cu1—Cu2—N1B	−10.37 (13)	Cu2—Cu1—N2A—C13A	28.9 (3)
O1A—Cu1—Cu2—N1B	170.07 (13)	O1B—Cu1—N2A—C9A	−152.9 (2)
N2A—Cu1—Cu2—N1B	−6.26 (16)	O1A—Cu1—N2A—C9A	54.4 (12)
N1A—Cu1—Cu2—N1B	179.75 (14)	N1A—Cu1—N2A—C9A	20.0 (2)
O21—Cu1—Cu2—N1B	81.33 (12)	O21—Cu1—N2A—C9A	118.6 (2)
O11—Cu1—Cu2—N1B	−102.11 (11)	O11—Cu1—N2A—C9A	−66.8 (2)
O1B—Cu1—Cu2—O12	89.42 (10)	Cu2—Cu1—N2A—C9A	−155.63 (16)
O1A—Cu1—Cu2—O12	−90.14 (10)	O1A—Cu2—N1B—C8B	−93.2 (6)
N2A—Cu1—Cu2—O12	93.53 (13)	O1B—Cu2—N1B—C8B	−144.97 (19)
N1A—Cu1—Cu2—O12	−80.45 (11)	N2B—Cu2—N1B—C8B	36.7 (2)
O21—Cu1—Cu2—O12	−178.88 (8)	O12—Cu2—N1B—C8B	127.29 (19)
O11—Cu1—Cu2—O12	−2.32 (8)	O22—Cu2—N1B—C8B	−63.2 (2)
O1B—Cu1—Cu2—O22	−88.43 (10)	Cu1—Cu2—N1B—C8B	−138.24 (17)
O1A—Cu1—Cu2—O22	92.01 (10)	O1A—Cu2—N1B—C7B	32.1 (7)
N2A—Cu1—Cu2—O22	−84.31 (13)	O1B—Cu2—N1B—C7B	−19.6 (2)
N1A—Cu1—Cu2—O22	101.70 (12)	N2B—Cu2—N1B—C7B	162.0 (2)
O21—Cu1—Cu2—O22	3.27 (8)	O12—Cu2—N1B—C7B	−107.4 (2)
O11—Cu1—Cu2—O22	179.83 (7)	O22—Cu2—N1B—C7B	62.1 (2)
O13—Cl1—O11—Cu1	−79.9 (2)	Cu1—Cu2—N1B—C7B	−12.9 (2)
O14—Cl1—O11—Cu1	159.15 (15)	O1A—Cu2—N2B—C13B	−31.5 (3)
O12—Cl1—O11—Cu1	40.37 (19)	O1B—Cu2—N2B—C13B	134.0 (11)
O1B—Cu1—O11—Cl1	−60.39 (16)	N1B—Cu2—N2B—C13B	155.6 (3)
O1A—Cu1—O11—Cl1	21.75 (15)	O12—Cu2—N2B—C13B	56.5 (3)
N2A—Cu1—O11—Cl1	−162.37 (16)	O22—Cu2—N2B—C13B	−117.8 (3)
N1A—Cu1—O11—Cl1	115.81 (17)	Cu1—Cu2—N2B—C13B	−30.6 (3)
O21—Cu1—O11—Cl1	−5.2 (4)	O1A—Cu2—N2B—C9B	150.9 (2)
Cu2—Cu1—O11—Cl1	−19.35 (14)	O1B—Cu2—N2B—C9B	−43.5 (13)
O13—Cl1—O12—Cu2	76.0 (2)	N1B—Cu2—N2B—C9B	−21.9 (2)
O14—Cl1—O12—Cu2	−164.11 (19)	O12—Cu2—N2B—C9B	−121.1 (2)
O11—Cl1—O12—Cu2	−44.6 (2)	O22—Cu2—N2B—C9B	64.6 (2)
O1A—Cu2—O12—Cl1	−14.02 (18)	Cu1—Cu2—N2B—C9B	151.84 (17)
O1B—Cu2—O12—Cl1	67.05 (18)	Cu1—O1A—C1A—C2A	131.2 (3)
N2B—Cu2—O12—Cl1	−117.38 (18)	Cu2—O1A—C1A—C2A	−11.7 (4)
N1B—Cu2—O12—Cl1	160.07 (18)	Cu1—O1A—C1A—C6A	−48.6 (3)
O22—Cu2—O12—Cl1	36.2 (5)	Cu2—O1A—C1A—C6A	168.5 (2)
Cu1—Cu2—O12—Cl1	26.51 (17)	O1A—C1A—C2A—C3A	178.1 (3)
O24—Cl2—O21—Cu1	−75.8 (2)	C6A—C1A—C2A—C3A	−2.1 (5)
O22—Cl2—O21—Cu1	44.1 (2)	C1A—C2A—C3A—C4A	2.3 (6)
O23—Cl2—O21—Cu1	163.3 (2)	C2A—C3A—C4A—C5A	−0.5 (7)
O1B—Cu1—O21—Cl2	13.46 (18)	C3A—C4A—C5A—C6A	−1.6 (7)
O1A—Cu1—O21—Cl2	−67.94 (18)	O1A—C1A—C6A—C5A	179.9 (3)
N2A—Cu1—O21—Cl2	116.36 (18)	C2A—C1A—C6A—C5A	0.1 (5)
N1A—Cu1—O21—Cl2	−161.18 (18)	O1A—C1A—C6A—C7A	1.2 (4)
O11—Cu1—O21—Cl2	−41.3 (4)	C2A—C1A—C6A—C7A	−178.6 (3)
Cu2—Cu1—O21—Cl2	−27.31 (17)	C4A—C5A—C6A—C1A	1.7 (6)
O24—Cl2—O22—Cu2	80.6 (2)	C4A—C5A—C6A—C7A	−179.6 (4)
O23—Cl2—O22—Cu2	−157.54 (17)	C1A—C6A—C7A—N1A	58.6 (4)
O21—Cl2—O22—Cu2	−38.9 (2)	C5A—C6A—C7A—N1A	−120.1 (3)
O1A—Cu2—O22—Cl2	58.62 (17)	C8A—N1A—C7A—C6A	175.9 (2)
O1B—Cu2—O22—Cl2	−23.06 (16)	Cu1—N1A—C7A—C6A	−63.1 (3)
N2B—Cu2—O22—Cl2	161.29 (17)	C7A—N1A—C8A—C9A	168.9 (2)
N1B—Cu2—O22—Cl2	−116.82 (18)	Cu1—N1A—C8A—C9A	44.1 (3)
O12—Cu2—O22—Cl2	8.1 (5)	C13A—N2A—C9A—C10A	−2.1 (5)
Cu1—Cu2—O22—Cl2	17.91 (15)	Cu1—N2A—C9A—C10A	−178.0 (3)
O1B—Cu1—O1A—C1A	−154.0 (2)	C13A—N2A—C9A—C8A	176.9 (3)
N2A—Cu1—O1A—C1A	−0.9 (12)	Cu1—N2A—C9A—C8A	1.0 (3)
N1A—Cu1—O1A—C1A	33.3 (2)	N1A—C8A—C9A—N2A	−30.5 (4)
O21—Cu1—O1A—C1A	−65.3 (2)	N1A—C8A—C9A—C10A	148.5 (4)
O11—Cu1—O1A—C1A	121.1 (2)	N2A—C9A—C10A—C11A	1.0 (6)
Cu2—Cu1—O1A—C1A	−153.7 (2)	C8A—C9A—C10A—C11A	−178.0 (4)
O1B—Cu1—O1A—Cu2	−0.29 (8)	C9A—C10A—C11A—C12A	1.2 (7)
N2A—Cu1—O1A—Cu2	152.8 (11)	C10A—C11A—C12A—C13A	−2.1 (7)
N1A—Cu1—O1A—Cu2	−173.02 (9)	C9A—N2A—C13A—C12A	1.2 (5)
O21—Cu1—O1A—Cu2	88.42 (8)	Cu1—N2A—C13A—C12A	176.3 (3)
O11—Cu1—O1A—Cu2	−85.20 (8)	C11A—C12A—C13A—N2A	1.0 (6)
O1B—Cu2—O1A—C1A	148.3 (2)	Cu1—O1B—C1B—C2B	10.3 (4)
N2B—Cu2—O1A—C1A	−32.8 (3)	Cu2—O1B—C1B—C2B	−130.8 (3)
N1B—Cu2—O1A—C1A	95.8 (6)	Cu1—O1B—C1B—C6B	−169.7 (2)
O12—Cu2—O1A—C1A	−124.1 (2)	Cu2—O1B—C1B—C6B	49.2 (3)
O22—Cu2—O1A—C1A	65.7 (2)	O1B—C1B—C2B—C3B	−178.7 (3)
Cu1—Cu2—O1A—C1A	148.0 (3)	C6B—C1B—C2B—C3B	1.3 (5)
O1B—Cu2—O1A—Cu1	0.29 (8)	C1B—C2B—C3B—C4B	−1.9 (6)
N2B—Cu2—O1A—Cu1	179.15 (9)	C2B—C3B—C4B—C5B	0.5 (6)
N1B—Cu2—O1A—Cu1	−52.3 (6)	C3B—C4B—C5B—C6B	1.5 (7)
O12—Cu2—O1A—Cu1	87.84 (9)	C4B—C5B—C6B—C1B	−2.1 (6)
O22—Cu2—O1A—Cu1	−82.38 (9)	C4B—C5B—C6B—C7B	179.1 (4)
O1A—Cu1—O1B—C1B	−146.2 (2)	O1B—C1B—C6B—C5B	−179.4 (3)
N2A—Cu1—O1B—C1B	36.0 (3)	C2B—C1B—C6B—C5B	0.6 (5)
N1A—Cu1—O1B—C1B	−90.1 (7)	O1B—C1B—C6B—C7B	−0.5 (4)
O21—Cu1—O1B—C1B	127.3 (2)	C2B—C1B—C6B—C7B	179.5 (3)
O11—Cu1—O1B—C1B	−64.3 (2)	C5B—C6B—C7B—N1B	119.9 (3)
Cu2—Cu1—O1B—C1B	−146.5 (3)	C1B—C6B—C7B—N1B	−58.9 (4)
O1A—Cu1—O1B—Cu2	0.29 (8)	C8B—N1B—C7B—C6B	−177.3 (2)
N2A—Cu1—O1B—Cu2	−177.50 (9)	Cu2—N1B—C7B—C6B	62.1 (3)
N1A—Cu1—O1B—Cu2	56.4 (7)	C7B—N1B—C8B—C9B	−168.9 (2)
O21—Cu1—O1B—Cu2	−86.17 (9)	Cu2—N1B—C8B—C9B	−43.9 (3)
O11—Cu1—O1B—Cu2	82.23 (8)	C13B—N2B—C9B—C10B	2.6 (5)
O1A—Cu2—O1B—C1B	152.1 (2)	Cu2—N2B—C9B—C10B	−179.7 (3)
N2B—Cu2—O1B—C1B	−13.7 (13)	C13B—N2B—C9B—C8B	−176.6 (3)
N1B—Cu2—O1B—C1B	−35.1 (2)	Cu2—N2B—C9B—C8B	1.2 (3)
O12—Cu2—O1B—C1B	64.0 (2)	N1B—C8B—C9B—N2B	28.9 (4)
O22—Cu2—O1B—C1B	−122.5 (2)	N1B—C8B—C9B—C10B	−150.3 (4)
Cu1—Cu2—O1B—C1B	152.4 (2)	N2B—C9B—C10B—C11B	−0.3 (6)
O1A—Cu2—O1B—Cu1	−0.29 (8)	C8B—C9B—C10B—C11B	178.8 (4)
N2B—Cu2—O1B—Cu1	−166.1 (11)	C9B—C10B—C11B—C12B	−2.6 (8)
N1B—Cu2—O1B—Cu1	172.48 (9)	C10B—C11B—C12B—C13B	3.1 (8)
O12—Cu2—O1B—Cu1	−88.38 (8)	C9B—N2B—C13B—C12B	−2.0 (5)
O22—Cu2—O1B—Cu1	85.08 (8)	Cu2—N2B—C13B—C12B	−179.4 (3)
O1B—Cu1—N1A—C8A	91.5 (7)	C11B—C12B—C13B—N2B	−0.9 (6)

### Hydrogen-bond geometry (Å, °)

**Table:**

*D*—H···*A*	*D*—H	H···*A*	*D*···*A*	*D*—H···*A*
N1A—H1AA···O23^i^	0.93	2.05	2.913 (4)	154
N1B—H1BA···O14^ii^	0.93	2.09	2.943 (4)	152
C7B—H7BA···O14^iii^	0.99	2.53	3.167 (4)	122
C22S—H22H···O13^iv^	0.98	2.47	3.264 (8)	138
C7A—H7AB···O23^v^	0.99	2.48	3.143 (4)	124
C4A—H4AA···N1S	0.95	2.69	3.643 (7)	175
C12B—H12B···N2S	0.95	2.67	3.616 (9)	171

Symmetry codes: (i) −*x*+1/2, *y*−1/2, −*z*+1/2; (ii) −*x*−1/2, *y*+1/2, −*z*+1/2; (iii) *x*, *y*+1, *z*; (iv) −*x*, −*y*+1, −*z*+1; (v) *x*, *y*−1, *z*.
